# Relationship of nutritional or systemic inflammatory markers with efficacy of gemcitabine and cisplatin with or without durvalumab therapy for patients with unresectable or metastatic biliary tract cancer: a retrospective study

**DOI:** 10.1186/s40780-026-00555-5

**Published:** 2026-02-16

**Authors:** Yayoi Fukushi, Kazuma Fujita, Yumiko Akamine, Haruka Igarashi, Katsuya Sasaki, Koji Fukuda, Hiroyuki Shibata, Masafumi Kikuchi

**Affiliations:** 1 Department of Pharmacy, Akita University Hospital, 1-1-1 Hondo, Akita, 010-8543 Japan; 2 Department of Clinical Oncology, Akita University, Akita, Japan

**Keywords:** Biliary tract cancer, Modified glasgow prognostic score, Prognostic factor, Gemcitabine, Cisplatin, Durvalumab

## Abstract

**Background:**

Nutritional or systemic inflammatory markers like modified Glasgow Prognostic Score (mGPS), prognostic nutritional index (PNI), neutrophil-lymphocyte ratio (NLR) and platelet-lymphocyte ratio (PLR) are useful prognostic indicators, but their role in patients with unresectable or metastatic biliary tract cancer receiving gemcitabine plus cisplatin (GC) or GC plus durvalumab is unclear. This study investigates the relationship of these markers with treatment outcomes and survival.

**Methods:**

Chemotherapy consisted of protracted infusion of gemcitabine (1000 mg/m^2^/day) and cisplatin (25 mg/m^2^/day) on day 1 and 8, or gemcitabine (1000 mg/m^2^/day), cisplatin (25 mg/m^2^/day) on day 1 and 8 and durvalumab (1500 mg/day 1) given once every 21 days. After six cycles of GC plus durvalumab therapy, durvalumab monotherapy was administered at 1500 mg every 4 weeks. PNI, NLR, PLR and mGPS were assessed before treatment using established cutoffs. Overall survival (OS) and time to treatment failure (TTF) were analyzed with Kaplan–Meier and Cox regression models. The primary endpoint was OS, defined as the time from the start of chemotherapy to death.

**Results:**

This study analyzed 103 patients with unresectable or metastatic biliary tract cancer treated with GC or GC plus durvalumab. For patients with PLR ≥ 148 the median survival was 13.7 months and for those with PLR < 148 the median OS was not estimated; this difference was significant (*p* = 0.016). For patients with mGPS 0–1 and 2 the median OS was 16.9 months and 7.9 months, respectively, and this difference was also significant (*p* < 0.001). Univariate analysis revealed that, among the nutritional or systemic inflammatory markers tested, mGPS 2 and PLR ≥ 148 significantly predicted shorter OS. Multivariate analysis further confirmed that mGPS 2 is an independent risk factor for shorter OS.

**Conclusion:**

A mGPS of 2 was a predictor of shorter OS for patients with unresectable or metastatic biliary tract cancer receiving GC or GC plus durvalumab. Evaluating mGPS prior to treatment initiation may support planning of individualized treatments that can enable clinicians to identify patients who are likely to benefit from chemotherapy and to exercise caution when considering systemic therapy for those with mGPS 2.

**Supplementary information:**

The online version contains supplementary material available at 10.1186/s40780-026-00555-5.

## Background

Biliary tract cancer is a malignant tumor that has a poor prognosis [1]. The different types of biliary tract cancer include intrahepatic biliary cancer, extrahepatic biliary cancer, gallbladder cancer, and ampullary cancer. The only curative treatment for biliary tract cancer is surgery, but since the disease is often diagnosed at an advanced stage patients often receive chemotherapy [1]. Based on the results of a randomized phase III trial (ABC-02 trial), a combination of gemcitabine and cisplatin (GC) therapy is used as a first-line treatment for patients with recurrent or unresectable biliary tract cancer [2]. The effectiveness of combining GC therapy with an immune checkpoint inhibitor (ICI) has also recently been demonstrated [3, 4]. The TOPAZ-1 trial demonstrated the efficacy and safety of GC therapy with additional durvalumab therapy [3] while the KEYNOTE-966 trial demonstrated the efficacy and safety of GC therapy with pembrolizumab [4]. ICI plus GC therapy is currently used as the first-line treatment for patients with unresectable or metastatic biliary tract cancer.

Nutritional assessment methods related to the prognosis of patients with cancer include those that combine serum nutritional indicators, inflammatory response indicators, and blood cell components, and serve as prognostic predictors for cancer patients [5–7]. In patients with biliary tract cancer, nutritional indicators such as the modified Glasgow Prognostic Score (mGPS), which combines albumin (Alb) and C-reactive protein (CRP) levels, the prognostic nutritional index (PNI) calculated from Alb and total lymphocyte count (TLC), the neutrophil-lymphocyte ratio (NLR) calculated from the absolute neutrophil count (ANC) and TLC, and the platelet-lymphocyte ratio (PLR) calculated from the platelet count and TLC have been shown to be prognostic predictors [8–10]. For patients with gastrointestinal cancers like metastatic pancreatic cancer or metastatic colorectal cancer, nutritional indicators measured before initiating chemotherapy are reported to be predictive of prognosis [11, 12]. However, few clinical studies have investigated the association between nutritional indicators and GC or GC plus durvalumab therapy efficacy for patients with unresectable or metastatic biliary tract cancer patients.

The aim of the present study was to clarify the clinical significance of nutritional and inflammation-based indices in assessing treatment outcomes by determining whether pretreatment PNI, NLR, PLR, and mGPS are associated with time to overall survival (OS) in patients with unresectable or metastatic biliary tract cancer receiving GC or GC plus durvalumab therapy.

## Methods

### Patients

This study was approved by the Ethics Committee of Akita University Graduate School of Medicine (No. 3067) and was conducted in accordance with the Declaration of Helsinki. Informed consent was obtained via an opt-out approach through the hospital website in accordance with institutional and ethical guidelines. Biliary tract cancer in all patients was diagnosed by physicians. Chemotherapy consisted of protracted infusion of gemcitabine (1000 mg/m^2^/day) and cisplatin (25 mg/m^2^/day) on day 1 and 8, or gemcitabine (1000 mg/m^2^/day), cisplatin (25 mg/m^2^/day) on day 1 and 8 and durvalumab (1500 mg/day 1) given once every 21 days. After six cycles of GC plus durvalumab therapy, durvalumab monotherapy was administered at 1500 mg every 4 weeks.

### Outcomes

The primary endpoint was OS, defined as the time from the start of chemotherapy to death. The secondary endpoint was time to treatment failure (TTF), defined as the time from the start of chemotherapy to treatment discontinuation or the estimated date of death. The treatment endpoint was defined as the date on which the treatment was deemed to have ended.

### Nutritional assessment methods

The PNI was calculated as 10 × Alb level (g/dL) + 0.005 × TLC (/mm^3^) [8]. The cutoff was set at PNI > 40 and PNI ≤ 40, as patients with PNI ≤ 40 are reported to have a poor prognosis [8].

The NLR was calculated based on the ANC and TLC. The cutoff was set at NLR ≥ 5 and NLR < 5, as patients with a NLR ≥ 5 are reported to have a poor prognosis [8].

The PLR was calculated based on the PLT and TLC. The cutoff was set at PLR ≥ 148 and PLR < 148, as patients with a PLR ≥ 148 are reported to have a poor prognosis [9].

For mGPS, patients with normal Alb (≥ 3.5 g/dL) and CRP (≤ 0.5 mg/dL) levels were given a score of 0. Those with only a low Alb (< 3.5 g/dL) or high CRP (> 0.5 mg/dL) level were given a score of 1, and those with both low Alb (< 3.5 g/dL) and high CRP (> 0.5 mg/dL) levels were given a score of 2 [7]. High mGPS is defined as mGPS ≥ 2 for multiple cancer types. As such, the cutoff was set at mGPS 0–1 and mGPS 2 [13, 14].

### Statistical analyses

The distribution of continuous variables was evaluated for normality using the Shapiro–Wilk test. Values for patient characteristics are presented as medians (range). Survival curves for the OS and TTF were analyzed using the Kaplan–Meier method with the log-rank test. Cox proportional hazard regression models were used to perform univariate and multivariate analyses. The objective variable was OS. In the multivariable model, age, sex, and primary tumor site, which are considered to be prognostic factors for biliary tract cancer, as well as nutritional or systemic inflammatory markers that showed a significant association with OS in the univariable analyses, were included as explanatory variables. A two-sided *P*-value < 0.05 was considered statistically significant. Statistical analyses were performed using SPSS 26.0 for Windows (SPSS IBM Japan Inc., Tokyo, Japan).

## Results

A total of 103 Japanese patients with biliary tract cancer treated with GC or GC plus durvalumab therapy and hospitalized between January 2010 and September 2023 were enrolled. The baseline characteristics for the 103 patients receiving GC or GC plus durvalumab therapy who were enrolled in this study are shown in Table 1. The patient population included 34 females and 69 males. The median (range) age and body surface area of the patients was 69 (26–80) years and 1.60 (1.18–1.98) m^2^, respectively. GC therapy and GC plus durvalumab therapy was given to 94 and 9 patients, respectively. The median (range) relative dose intensity for gemcitabine and cisplatin was 63.3% (26.7%-100%) and 60.8% (25.3%-88.8%), respectively. The median (range) for the average relative dose intensity was 66.5% (26.5%-90.0%). The median (range) values for PNI, NLR and PLR were 40.1 (27.9–89.5), 3.4 (0.4–24.5) and 200 (27–671.7), respectively. For mGPS, 36, 28, and 39 patients had a score of 0, 1, and 2, respectively.

**Table 1:** Patient clinical characteristics

Sex (female : male)	34:69	
Age (years)	69 (26–80)	
BSA (m^2^)	1.60 (1.18–1.98)	
Unresectable or recurrent	74:29	
Performance Status (0 : 1 : ≥ 2)	87:13:3	
Primary cancer site		
Intrahepatic biliary cancer	29	
Extrahepatic biliary cancer	44	
Gallbladder cancer	23	
Duodenal papilla cancer	7	
Disease classification (Locally advanced : Metastatic)		27:76
Chemotherapy regimen (GC: GC plus Dur)	94:9	
RDI of gemcitabine (%)	63.3 (26.7–100.0)	
RDI of cisplatin (%)	60.8 (25.3–88.8)	
ARDI (%)	66.5 (26.5–90.0)	
Laboratory test values		
White blood cells (/μL)	5500 (2200–16700)	
Neutrophils (/μL)	3600 (1232–14700)	
Lymphocytes (/μL)	1080 (280–9300)	
Hemoglobin (g/dL)	11.7 (6.0–16.4)	
Platelets (×10^4^/μL)	24.3 (10.2–49.8)	
Aspartate transaminase (IU/L)	29(11–231)	
Alanine transaminase (IU/L)	28 (8–226)	
Total bilirubin (mg/dL)	0.6 (0.2–4.3)	
Serum creatinine (mg/dL)	0.68 (0.33–1)	
Albumin (g/dL)	3.6 (2.2–4.8)	
C-reactive protein (mg/dL)	0.83 (0.02–23.9)	
Prognostic nutritional index	40.1 (27.9–89.5)	
Neutrophil-lymphocyte ratio	3.4 (0.4–24.5)	
Platelet-lymphocyte ratio	200 (27–671.7)	
Modified Glasgow prognostic score (0 : 1 : 2)	36:28:39	

Variables are presented as number or median (minimum—maximum)

BSA: Body surface area; GC: gemcitabine plus cisplatin; Dur: Durvalumab; RDI: Relative dose intensity; ARDI: Average relative dose intensity

**Fig. 1:**
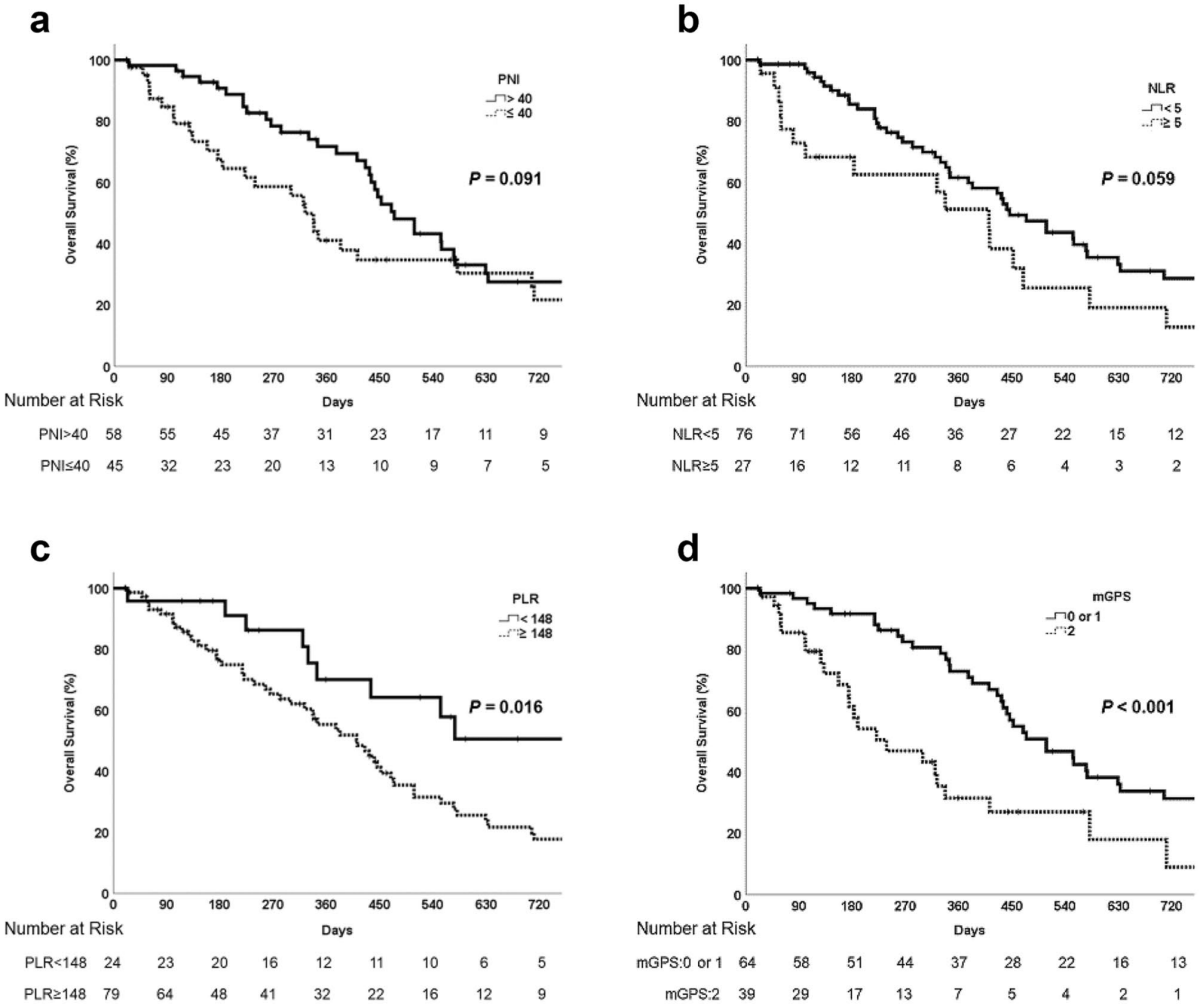
Overall survival in patients with unresectable or metastatic biliary tract cancer receiving gemcitabine plus cisplatin (GC) with or without durvalumab based on (**a**) prognostic nutritional index (PNI; >40 vs. ≤40), (**b**) neutrophil-to-lymphocyte ratio (NLR; < 5 vs. ≥ 5), (**c**) platelet-to-lymphocyte ratio (PLR; < 148 vs. ≥ 148), and (**d**) modified Glasgow prognostic score (mGPS; 0–1 vs. 2)

The median (range) OS among all patients was 14.5 (0.8–58.3) months. For patients with PNI > 40 and PNI ≤ 40 the median OS was 15.8 months and 10.7 months, respectively, but this difference was not significant (*p* = 0.091) (Fig. 1a). For patients with NLR < 5 and NLR ≥ 5 the median OS was 14.9 months and 13.7 months, respectively, and this difference was also not significant (*p* = 0.059) (Fig. 1b). For patients with PLR < 148 the median OS was not estimated and for those with PLR ≥ 148 the median OS was 13.7 months; this difference was significant (*p* = 0.016) (Fig. 1c). For patients with mGPS 0–1 and 2 the median OS was 16.9 months and 7.9 months, respectively, and this difference was significant (*p* < 0.001) (Fig. 1d).

**Fig. 2:**
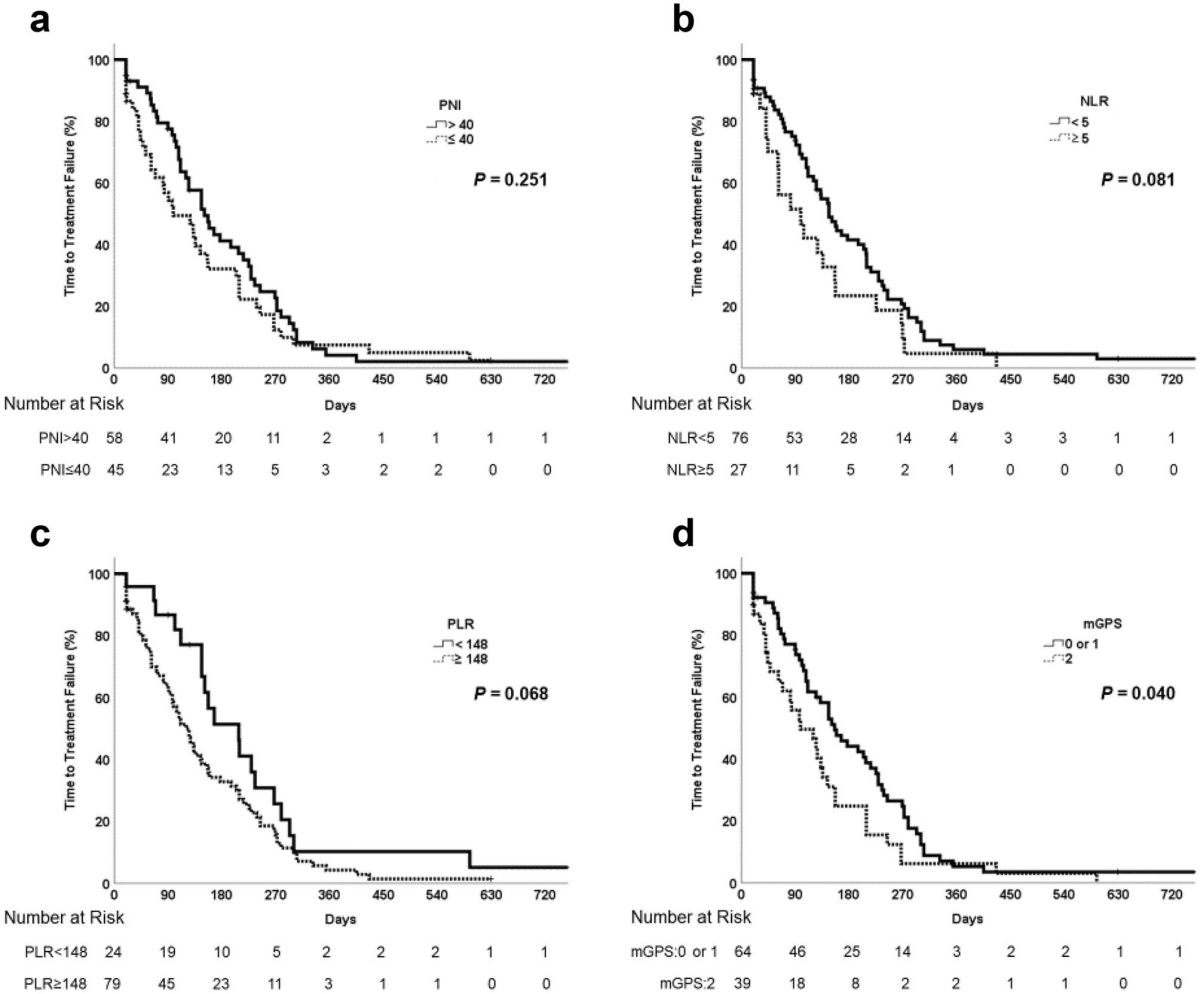
Time to treatment failure in patients with unresectable or metastatic biliary tract cancer receiving gemcitabine plus cisplatin (GC) with or without durvalumab, based on (**a**) prognostic nutritional index (PNI; > 40 vs. ≤ 40), (**b**) neutrophil-to-lymphocyte ratio (NLR; < 5 vs. ≥ 5), (**c**) platelet-to-lymphocyte ratio (PLR; < 148 vs. ≥ 148), and (**d**) modified Glasgow prognostic score (mGPS; 0–1 vs. 2)

The median (range) TTF among all patients was 4.5 (0.7–25.4) months. For patients with PNI > 40 and PNI ≤ 40 the median TTF was 5.0 months and 3.3 months, respectively, but this difference was not significant (*p* = 0.251) (Fig. 2a). For patients with NLR < 5 and NLR ≥ 5 the median TTF was 4.9 months and 3.3 months, respectively, but this difference was not significant (*p* = 0.081) (Fig. 2b). For patients with PLR < 148 and PLR ≥ 148 the median TTF was 6.9 months and 4.0 months, respectively, and this difference also was not significant (*p* = 0.068) (Fig. 2c). For patients with mGPS 0–1 and 2 the median TTF significantly differed at 5.2 months and 3.0 months, respectively (*p* = 0.040) (Fig. 2d).

Univariate Cox proportional hazard regression analyses of OS was next performed using sex, age, recurrent, extrahepatic biliary cancer, metastatic disease classification, chemotherapy regimen, PNI, NLR, PLR and mGPS as variables (Table 2). Extrahepatic biliary cancer (HR 0.341, 95% CI 0.191–0.609, *p* < 0.001), mGPS 2 (HR 1.578, 95% CI 1.206–2.066, *p* < 0.001) and PLR ≥ 148 (HR 2.331, 95% CI 1.143–4.750, *p* = 0.002) were statistically significant predictors of OS, but PNI ≤ 40 (HR 1.559, 95% CI 0.927–2.620, *p* = 0.094), NLR ≥ 5 (HR 1.730, 95% CI 0.972–3.081, *p* = 0.062) were not.

**Table 2:** Univariate cox proportional hazard regression analyses for overall survaival

	HR	95% CI	*P*-value
Sex male	0.878	0.510–1.512	0.640
Age	0.993	0.972–1.015	0.522
Recurrent	0.855	0.490–1.492	0.581
Extrahepatic biliary cancer	0.341	0.191–0.609	<0.001*
Desease classification Metastatic	1.346	0.763–2.375	0.305
Chemothrapy regimen GC	0.488	0.218–1.093	0.081
PNI ≤ 40	1.559	0.927–2.620	0.094
NLR ≥ 5	1.730	0.972–3.081	0.062
PLR ≥ 148	2.331	1.143–4.750	0.002*
mGPS 2	1.578	1.206–2.066	<0.001*

GC, gemcitabine plus cisplatin; Dur, Durvalumab; PNI, Prognostic nutritional index; NLR, Neutrophil-lymphocyte ratio; PLR, Platelet-lymphocyte ratio; mGPS, modified Glasgow prognostic score; HR, hazard ratio; CI, confidence interval

*Statistically significant

In a multivariate Cox proportional hazard regression analysis, mGPS 2 remained a risk factor that influenced OS of patients receiving GC or GC plus durvalumab therapy (Table 3). A multivariable Cox proportional hazards regression analysis was performed with PLR as the main explanatory variable. After adjusting for age, sex, and primary tumor site, PLR ≥ 148 was significantly associated with OS; however, this association was no longer significant after additional adjustment for mGPS (Table 4). For patients with mGPS 0–1 and 2 receiving GC therapy, the median OS was 18.4 months and 7.9 months, respectively, and this difference was significant (*p* = 0.002) (Supplementary Figure 1). On the other hand, for patients with mGPS 0–1 and 2 receiving GC plus durvalumab therapy the median OS was 13.7 months and 6.1 months, respectively, but the difference was not significant (*p* = 0.175) (Supplementary Figure 2). For patients with mGPS 0–1 and 2 receiving GC therapy the median TTF was 5.2 months and 3.3 months, respectively, and this difference was significant (*p* = 0.046) (Supplementary Figure 3). For patients with mGPS 0–1 and 2 receiving GC plus durvalumab therapy the median TTF was 4.9 months and 1.4 months, respectively, but the difference was not significant (*p* = 0.707) (Supplementary Figure 4).

**Table 3:** Multivariate Cox proportional hazard regression analysis of the association between the mGPS and overall survival

	Overall Survival		
Variable	HR	95% CI	*P*-value
Crude (mGPS 2)	1.578	1.206–2.066	0.001*
Adjusted for age and sex	1.578	1.206–2.066	0.001*
Adjusted for age, sex, and primary cancer site	1.449	1.103–1.902	0.008*
Adjusted for age, sex, primary cancer site and PLR	1.427	1.082–1.881	0.012*

mGPS, modified Glasgow prognostic score; PLR, Platelet-lymphocyte ratio; HR, hazard ratio; CI, confidence interval

*Statistically significant

**Table 4:** Multivariate Cox proportional hazard regression analysis of the association between the PLR and overall survival

	Overall Survival		
Variable	HR	95% CI	*P*-value
Crude (PLR ≥ 148)	2.331	1.143–4.750	0.002*
Adjusted for age and sex	2.331	1.143–4.750	0.002*
Adjusted for age, sex, and primary cancer site	2.105	1.029–4.303	0.041*
Adjusted for age, sex, primary cancer site and mGPS	2.015	0.980–4.140	0.057

mGPS, modified Glasgow prognostic score; PLR, Platelet-lymphocyte ratio; HR, hazard ratio; CI, confidence interval

*Statistically significant

## Discussion

This study examined the association between OS and mGPS of patients with unresectable or metastatic biliary tract cancer who received GC or GC plus durvalumab therapy. Patients with biliary tract cancer receiving GC or GC plus durvalumab who had mGPS 2 had significantly shorter OS relative to patients with mGPS 0–1. On the other hand, PNI, NLR, and PLR were not associated with OS.

Okuno et al. reported that patients with unresectable hilar cholangiocarcinoma had a median survival time (MST) of 9.2 months and those with mGPS 1–2 had a significantly shorter MST than patients with mGPS 0 [8]. From their multivariate analysis results, Okuno et al. concluded that mGPS 1 and mGPS 2 are independent predictors of shorter MST [8]. Okuno et al. included only patients with perihilar cholangiocarcinoma who underwent surgery, but, like this study, found that mGPS is related to prognosis. Our study included only patients who received GC therapy, the standard chemotherapy for biliary tract cancer, and the results were consistent with those reported by Okuno et al. These findings suggest that patients with biliary tract cancer and an mGPS of 2 may have a poor prognosis regardless of whether they undergo chemotherapy. However, the potential influence of chemotherapy on these results remains unclear. Therefore, additional studies focusing exclusively on patients who have not received chemotherapy are warranted to further validate our findings. In addition, a meta-analysis by Zhou et al. showed that high mGPS was a predictive factor for shorter OS and disease-free survival/recurrence-free survival in patients with biliary tract cancer [15]. The Zhou et al. report suggests that high mGPS is a predictor of prognosis in patients with biliary tract cancer, and is similar to the results of the present study, which only included patients with biliary tract cancer who received chemotherapy. Yamamoto et al. showed that patients with breast cancer who received chemotherapy and had mGPS 2 had a significantly shorter TTF compared with patients with mGPS of 0 or 1 [16]. High mGPS has been shown to be a poor prognostic factor even in patients undergoing ICI [17]. Jonass et al. analyzed the effectiveness of treatment beyond progression (TBP) in patients with urothelial carcinoma, renal cell carcinoma, or non-small cell lung cancer treated with ICI and found that a lower mGPS at PD was associated with prolonged survival in all of these cancer types [17]. Higher mGPS was also shown to be associated with shorter survival times in Japanese non-small cell lung cancer patients treated with ICI therapy [13]. These reports are consistent with the findings of the present study, which, to our knowledge, is the first to examine the prognostic value of mGPS for patients with biliary tract cancer who received chemotherapy. Together, these reports indicate that patients with high mGPS who receive chemotherapy have a worse prognosis, regardless of cancer type or type of drug therapy. In support of these results, in our study the different treatment regimens (GC or GC plus durvalumab therapy) were similarly affected by mGPS.

On the other hand, here we showed that PNI, NLR and PLR were not associated with OS. Okuno et al. reported that NLR ≥ 5 is a predictor of shorter OS in patients with biliary tract cancer [8]. However, the Okuno et al. study only included patients with perihilar cholangiocarcinoma and in our cohort only 24 patients had this condition. As such, the association between NLR and OS may differ depending on the primary cancer site. Indices like NLR, PLR, and PNI mainly reflect immune or hematologic conditions. Meanwhile, the mGPS value combines serum CRP and Alb levels, and thereby reflects both systemic inflammation and nutritional depletion more comprehensively than other indices. Decreased Alb and elevated CRP levels have been reported to be risk factors for severe acute cholangitis [18]. In patients with pancreatic cancer who underwent preoperative chemotherapy, interruption or postponement of treatment due to cholangitis has been shown to shorten OS [19]. Therefore, these factors may also contribute to treatment interruption in patients with biliary tract cancer receiving chemotherapy, thereby leading to shorter OS. Ogul et al. reported that PNI and NLR were useful predictors of 6-month PFS and 12-month OS in patients with advanced biliary tract cancer who received GC therapy [10]. No consensus exists regarding cutoff values for these indices, and these differences may therefore account for the inconsistent results across studies. Further studies are needed to establish standardized cutoff values.

This study has several limitations that should be taken into account when interpreting the findings. First, GC plus ICI therapy is currently the standard first-line treatment for unresectable or metastatic biliary tract cancer [3, 4]. In the present study, only approximately 10% (9/103) of patients received GC plus durvalumab; therefore, further investigations focusing on patients treated with GC plus ICIs are warranted. Second, this was a retrospective, single-center study with a relatively small sample size, which may limit the generalizability and statistical power of the findings. Hence, additional prospective multicenter studies with larger cohorts are needed to validate our results. However, the univariate and multivariate analyses of the present study identified only mGPS 2 as an independent factor associated with a shorter OS after GC or GC plus durvalumab therapy for patients with biliary tract cancer. These findings suggest that evaluating mGPS before initiating chemotherapy could help identify patients who are more likely to benefit from systemic treatment. Specifically, in our study, patients with an mGPS 0–1 had a median OS of 16.9 months, whereas in the TOPAZ-1 trial, patients with unresectable or metastatic biliary tract cancer who received GC plus durvalumab therapy had a median OS of 12.8 months [3]. These findings suggest that patients with an mGPS of 0–1 are more likely to benefit from chemotherapy. Therefore, these results may help patients and clinicians make informed decisions at the start of treatment. On the other hand, in our study, the median OS was only 7.9 months in patients with mGPS 2, indicating that chemotherapy should be initiated with caution in this group.

Among various inflammation- and nutrition-based indices, only mGPS 2 was identified as an independent prognostic factor in this study. In contrast to other indices like NLR, PLR, and PNI, which mainly reflect immune or hematologic conditions, mGPS combines CRP and Alb levels and may therefore more accurately represent both systemic inflammation and nutritional depletion.

## Conclusion

The present study demonstrated that an mGPS of 2 independently predicts shorter OS among patients with unresectable or metastatic biliary tract cancer receiving GC or GC plus durvalumab therapy. Compared with other inflammatory and nutritional markers like NLR, PLR, and PNI, mGPS appears to offer a more comprehensive reflection of systemic inflammation and cancer-related cachexia. Evaluating mGPS prior to treatment initiation may support individualized treatment planning, enabling clinicians to identify patients who are likely to benefit from chemotherapy and to exercise caution when considering systemic therapy for those with mGPS 2.

## Electronic supplementary material

Below is the link to the electronic supplementary material.

Supplementary Material 1

Supplementary Material 2

Supplementary Material 3

Supplementary Material 4

## Data Availability

No datasets were generated or analysed during the current study.

## Abbreviations

Alb: Albumin
ANC: Absolute neutrophil count
CRP: C-reactive protein
GC: Gemcitabine and cisplatin
ICI: Immune checkpoint inhibitor
mGPS: Modified Glasgow Prognostic Score
MST: Median survival time
NLR: Neutrophil-lymphocyte ratio
OS: Overall survival
PLR: Platelet-lymphocyte ratio
PNI: Prognostic nutritional index
TBP: Treatment beyond progression
TLC: Total lymphocyte count
TTF: Time to treatment failure
