# Supplementary material for: Relationship of nutritional or systemic inflammatory markers with efficacy of gemcitabine and cisplatin with or without durvalumab therapy for patients with unresectable or metastatic biliary tract cancer: a retrospective study
Source: J Pharm Health Care Sci. 2026 Feb 16;12:33. doi: 10.1186/s40780-026-00555-5 (PMC12983643; doi:10.1186/s40780-026-00555-5)
Supplement: Supplementary file 1 — Supplementary Material 1 [file 40780_2026_555_MOESM1_ESM.pptx]

## Slide 1
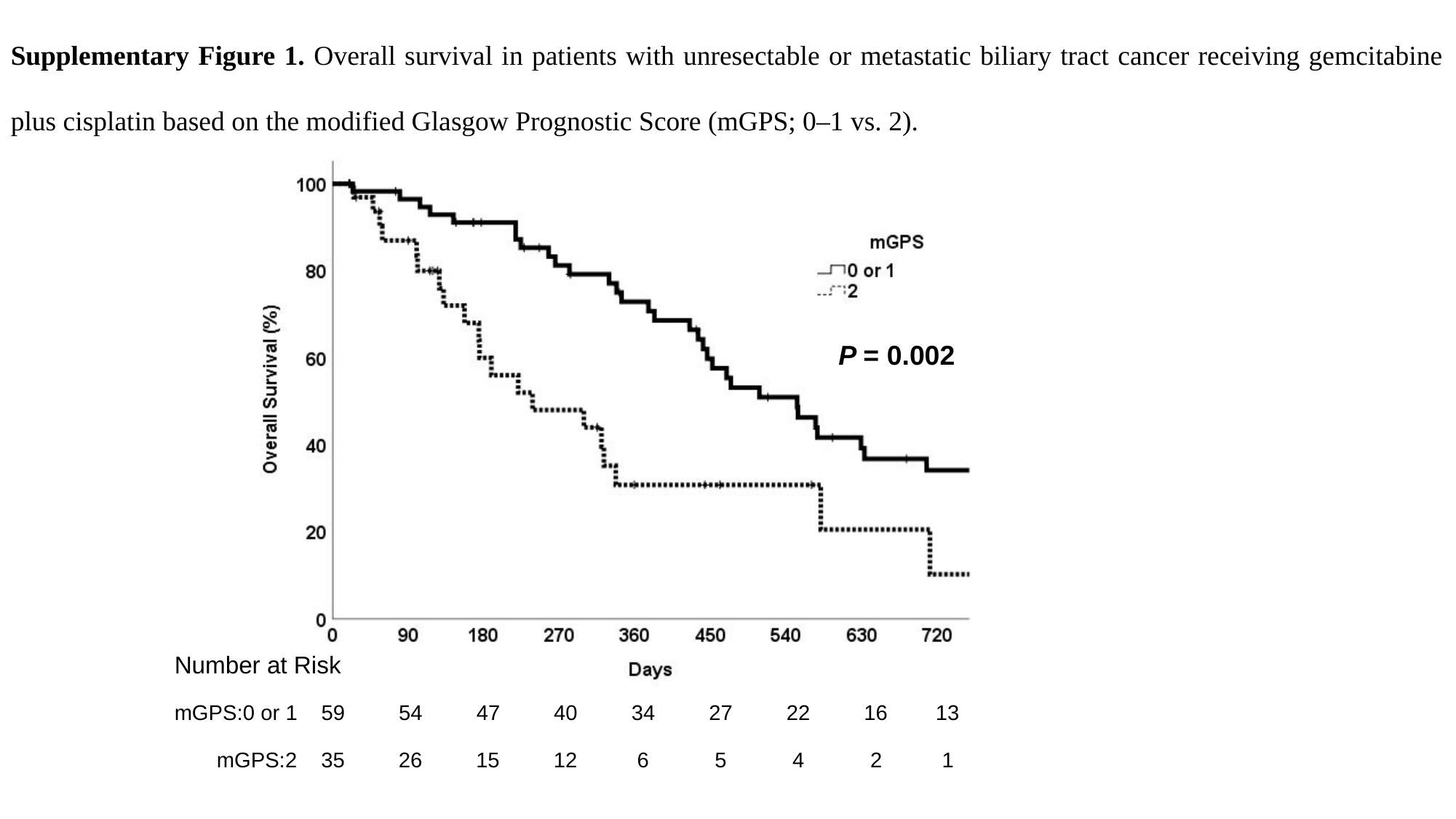

Supplementary Figure 1. Overall survival in patients with unresectable or metastatic biliary tract cancer receiving gemcitabine plus cisplatin based on the modified Glasgow Prognostic Score (mGPS; 0–1 vs. 2).
 P = 0.002
Number at Risk
mGPS:0 or 1 59 54 47 40 34 27 22 16 13
mGPS:2 35 26 15 12 6 5 4 2 1
