# Supplementary material for: Relationship of nutritional or systemic inflammatory markers with efficacy of gemcitabine and cisplatin with or without durvalumab therapy for patients with unresectable or metastatic biliary tract cancer: a retrospective study
Source: J Pharm Health Care Sci. 2026 Feb 16;12:33. doi: 10.1186/s40780-026-00555-5 (PMC12983643; doi:10.1186/s40780-026-00555-5)
Supplement: Supplementary file 4 — Supplementary Material 4 [file 40780_2026_555_MOESM4_ESM.pptx]

## Slide 1
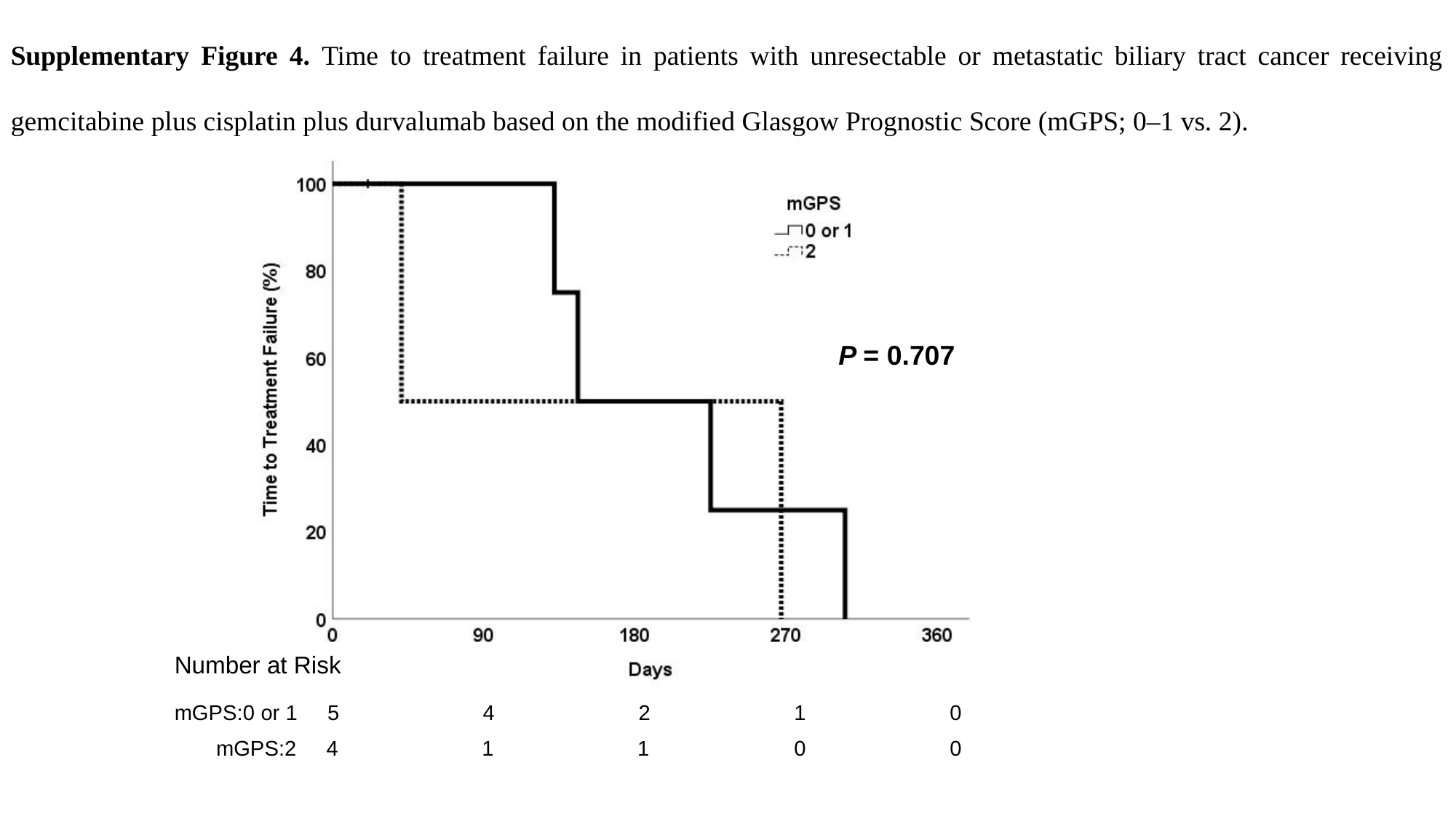

Supplementary Figure 4. Time to treatment failure in patients with unresectable or metastatic biliary tract cancer receiving gemcitabine plus cisplatin plus durvalumab based on the modified Glasgow Prognostic Score (mGPS; 0–1 vs. 2).
 P = 0.707
Number at Risk
mGPS:0 or 1 5 4 2 1 0
mGPS:2 4 1 1 0 0
